# Prognostic value and chemotherapy response prediction of a proliferation essential gene signature in colon cancer

**DOI:** 10.1042/BSR20230733

**Published:** 2023-07-19

**Authors:** Jinsheng Liu, Wei Liang, Yanqin Xu, Shishun Zhong

**Affiliations:** 1 Department of Gastrointestinal Surgery, Provincial Clinic Medical College, Fujian Medical University, Fujian Provincial Hospital, Fujian Province, China; 2 Department of Digestive Endoscopy, Provincial Clinic Medical College, Fujian Medical University, Fujian Provincial Hospital, Fujian Province, China

**Keywords:** chemotherapy response, Colon cancer, DepMap, prognosis, Proliferation

## Abstract

**Background:** Colon cancer is a common malignant tumor in the digestive tract. Exploring new treatment targets is of great significance for improving the survival rate of colon cancer patients. The present study mainly analyzes the impact of proliferation essential genes (PLEGs) on the prognosis and chemotherapy response of colon cancer patients, as well as identifying the expression and cellular functions of important PLEG.

**Methods:** The DepMap database was utilized for identification of PLEG in colon cancer cells. Through DEGs screening, WGCNA, univariate cox regression survival analysis, and LASSO, a PLEG signature (PLEGs) model was constructed. The impact of PLEGs on the prognosis of colon cancer patients and their response to chemotherapy was further analyzed. Finally, we conducted a random forest analysis and implemented functional experiments to investigate the prominent PLEG that is linked to the development of colon cancer.

**Results:** Based on the expression and prognosis of PLEG, we constructed a PLEGs prognosis model which can effectively predict the prognosis of colon cancer patients and their response to chemotherapy treatment. Random forest analysis showed that *UBA1* is a key PLEG in the progression of colon cancer. Immunohistochemistry results revealed that UBA1 protein is significantly upregulated in colon cancer tissues. Cell functional experiments demonstrated that knocking down *UBA1* can inhibit the proliferation, invasion, and migration abilities of colon cancer cells.

**Conclusion:** PLEGs have the potential to serve as predictive biomarkers for prognosis and chemotherapy response in colon cancer patients. Among the PLEG, *UBA1* plays a prominent role in promoting the malignant progression of colon cancer cells.

## Introduction

Colorectal cancer (CRC) is a malignant tumor of the digestive tract that occurs in the colorectum, and its incidence ranks among the top five in both men and women [1]. Colon cancer is the most common pathological type of colorectal cancer. The main approach for treating colon cancer patients is through surgery coupled with adjuvant chemotherapy. To combat this disease, primary chemotherapeutic drugs including oxaliplatin, 5-fluorouracil, and capecitabine are utilized. Sadly, the recurrence and onset of multidrug resistance often lead to a dwindling survival rate among these patients [2–4]. Scientists have shown that there are various processes implicated in the inception, progression, propagation, and spread of colon cancer, such as the elevation of oncogenic genes and the reduction of tumor suppressive genes. Nevertheless, the exact mechanism responsible for the formation of colon cancer in humans is still ambiguous. Further investigations will assist in enhancing our knowledge of colon cancer and pinpointing prospective targets for novel therapies.

Cancer develops due to a series of genomic alterations that accumulate over time. These alterations include single-nucleotide mutations, insertions and deletions of DNA bases, changes in the number of copies of genes, and abnormal translocation of chromosomes [5–8]. These alterations ultimately lead to the dependency of tumor cells on specific signaling pathways or genes that promote their growth and proliferation. Identifying genes that are dysregulated in cancer and developing therapies to target them has been made possible by advances in genomic sequencing technologies. By utilizing high-throughput genetic screening techniques to modulate gene expression at the transcriptional level, it is possible to identify genes that regulate cellular growth and proliferation. Even if these genes are not mutated in tumor cells themselves, they may still play a critical role in promoting tumor cell proliferation and could thus be exploited for targeted cancer treatments. To comprehensively assess gene dependencies in cancer cells, the Broad and Sanger Institutes conducted genome-wide CRISPR-KO screenings in hundreds of cancer cell lines. Their collaborative efforts led to the creation of the DepMap database, which contains their screening results and is designed to promote data sharing and knowledge exchange in the field of cancer research [9].

This study utilized the DepMap database to identify genes essential for the proliferation of colon cancer cells, which were then used to develop a reliable prognostic model for overall survival. The model was developed through differential gene expression screening, weighted gene correlation network analysis (WGCNA), univariate cox regression survival analysis, and least absolute shrinkage and selection operator (LASSO) analysis. The resulting proliferation essential genes signature (PLEGs) model proved effective in predicting overall survival and chemotherapy response among colon cancer patients. Analysis using random forest method identified *UBA1* as a key gene associated with colon cancer progression, and this finding was confirmed in tumor tissue samples using Immunohistochemistry (IHC) analysis. Finally, the study evaluated the effects of *UBA1* knockdown on colon cancer cell proliferation, invasion, and migration.

## Methods

### Identifying proliferation essential genes (PLEG) of colon cancer through DepMap database

Cancer Dependency Map (DepMap, https://depmap.org/portal/) is a database containing the expression, mutation, copy number of genes in more than 1000 tumor cell lines, and information about gene dependency in more than 500 tumor cell lines. In the study, DepMap database was used to identify the gene dependence of colon cancer cell lines. CERES is a parameter to assess the degree of gene necessity. If the CERES score of a gene was a negative number, it means that knocking out the gene could suppress the proliferation and survival of cells. Genes with CERES score less than −1 in more than 75% of colon cancer cell lines were defined as proliferation essential genes in colon cancer [10].

### Gene expression cohort retrieval and different expression genes (DEGs) analysis

Based on the public Gene expression omnibus (GEO) and TCGA (The Cancer Genome Atlas) databases, seven expression data related to colon cancer were obtained, including GSE87211 [11] (203 colon cancer tissues with long-term follow-up and 160 normal tissues), GSE110225 [12] (13 colon cancer tissues and 13 normal tissues), GSE9348 [13] (70 colon cancer tissues and 12 normal tissues), GSE103512 [14] (55 colon cancer tissues and 14 normal tissues), GSE106582 [15] (77 colon cancer tissues and 117 normal tissues), GSE39582 (345 patients not treated with chemotherapy and 240 patients treated with chemotherapy), and TCGA-COAD cohort (456 colon cancer tissues and 41 normal tissues). The R ‘limma’ package was performed to identify the DEGs between the colon cancer group and normal group using GSE87211 database, genes with a |log2FC| ≥ 0.585 and FDR < 0.05 were considered significant DEGs.

### KEGG and Gene Ontology enrichment analysis

In the study, the DAVID database (https://david-d.ncifcrf.gov/) was used to perform the functional annotation for DEGs. KEGG pathway and Gene Ontology (GO) enrichment analysis for the DEGs were performed using DAVID database [16].

### Gene set enrichment analysis (GSEA) analysis

GSEA was performed by using GSEA software (http://software.broadinstitute.org/gsea/) [17]. Each colon cancer patient was divided into the low expression of *UBA1* group (50%) and the high expression of *UBA1* group (50%) to identify related pathways. A *p* value of <0.05 was considered statistically significant.

### Gene set variation analysis (GSVA) analysis

The GSVA analysis was performed by using R ‘GSVA’ package [18]. GSVA was used to detect cell cycle-related pathway activity changes in colon cancer samples.

### LASSO analysis

LASSO was performed to construct a proliferation essential genes signature (PLEGs) model with the help of R ‘survival’ and ‘glmnet’ package. LAASO was developed by analyzing the transcriptomic data of proliferation essential genes to establish a PLEGs module: PLEGs = (−0.7350 × expression level of *TWISTNB*) + (−0.725 × expression level of *ESPL1*) + (−0.159 × expression level of *TOPBP1*) + (−0.386 × expression level of *CPSF3*) + (0.375 × expression level of *UBA1*) + (0.579 × expression level of *SEC61A1*).

### Weighted gene correlation network analysis (WGCNA)

WGCNA was performed on the transcriptomic data of proliferation essential genes using the WGCNA R package to create networks of signed co-expressed gene modules [19]. A soft-threshold parameters of *β* = 6 to the power was chosen, a signed network was used, a minimum cluster size was used for dendrogram clustering detection, and a total of three modules were created. The grey module was used for unassigned genes and did not represent a real module.

### Colon cancer patients

A total of 30 colon cancer specimens were obtained from Fujian Provincial Hospital. The study was performed with the approval of the Ethics Committee of Fujian Provincial Hospital and complied with the Helsinki Declaration. The written informed consent was obtained from all patients.

### IHC staining analysis

IHC staining was used to measure the protein expression of UBA1 in colon cancer tissues and normal tissues. Slides were incubated with anti-UBA1 (67198-1-Ig, Proteintech, Wuhan, China, diluted 1:400). The IHC scores of UBA1 protein were evaluated by two independent pathologists. The staining intensity score was calculated as followed: 0 score, no staining; 1 score, weak staining; 2 score, moderate staining; and 3 score, strong staining. The percentage of stained positive cells was calculated as followed: 1 score, 0–25%; 2 score, 26–50%; 3 score, 51–75% and 4 score, 75–100%. The score of positive tumor cells and the staining intensity were added to produce a weighted score for each case.

### Cells culture and transfection

HCT116 and DLD1 were obtained from American Type Culture Collection (ATCC, Manassas, VA, U.S.A.). HCT116 cell line was isolated from the colon of an adult male with colon cancer. DLD-1 is a colorectal adenocarcinoma cell line isolated from the large intestine of a colon adenocarcinoma patient. Cells were cultured in a mixed medium with half F12 (BI, China) and DMEM containing 10% fetal bovine serum (FBS) and 100 U/mL penicillin and 0.1 mg/ml streptomycin at 37°C with 5% CO_2_. The sequence of shRNAs targeting *UBA1* was cloned into the pLVX vector. The sequence of *UBA1* shRNA1 was 5′-CAGGGCATGGTTGAACTCAACGGAA-3′; The sequence of *UBA1* shRNA2 was 5′-CAAAGTCCTGGGTCCTTATACCTTT-3′. The transfection was performed using lipofectamine 2000 according to the manufacturer’s guidelines.

### RNA isolation and RT-qPCR

Total RNA was extracted using TRIzol. mRNA Reverse Transcription Kit was performed for the reverse transcription. Real-time PCR (RT-PCR) was performed using SYBR Green Kit. The primer sequences were shown as follows: *UBA1* forward primer 5′-GAGCGGGGACTTTGTCTCCT-3′; *UBA1* reverse primer 5′-CTTTGACCTGACTGACGAT-3′. *GAPDH* forward primer 5′-GGAAGGACTCATGACCACAGTCC-3′; *GAPDH* reverse primer 5′-TCGCTGTTGAAGTCAGAGGAGACC-3′. *GAPDH* was used as the control. *UBA1* expression was determined by the 2^−ΔΔCT^ method.

### CCK-8 assay

CCK-8 assay was used to measure the proliferation of cells. Cells were cultured in four 96-well plates for 0, 24, 48, and 72 h. Before absorbance measuring, 10 µl of CCK-8 solution was added to the plates and incubated at 37°C for 2 h. Finally, the absorbance was measured at 450 nm with a microplate reader.

### Wound-healing assay

Wound-healing assay was performed to evaluate the migration capacity of cells. Cells (3 × 10^6^ cells/well) were cultured in six-well plates and aggregated to about 90%. PBS solution was performed to wash the dislodged cellular debris away at least 3 times before scratching. Next, a 10 µl of spear made use of scratching wounds, and PBS solution was used to wash the dislodged cellular debris away. Meanwhile, cells were cultured in serum-free medium at 37°C with 5% CO_2_ for 48 h and filmed microscopically again.

### Trans-well assay

Trans-well assay was performed to evaluate the invasion capacity of cells. Cells were cultured in 24-well plates with a 300 µl of serum-free medium and an invasion chamber. The underlying chamber was added 500 µl of complete medium and cultured at 37°C with 5% CO_2_ for 48 h. The invaded cells passing through the membrane were fixed with PBS: methanol (3:7) solution and stained with 500 µl of 0.1% crystal violet for about 20 min. Cells on the overhead chamber surface with cotton swabs to wipe off, the quantity of cells were photographed stochastically at six spotting areas.

### Statistical analysis

Each experiment was repeated in triplicate at least three times. Student’s *t*-test was performed using GraphPad Prism. Survival curves were estimated by the Kaplan–Meier method with the log-rank test. For CCK8 assay, two-way ANOVA was calculated using GraphPad Prism. All *P-*values < 0.05 were considered statistically significant.

## Results

### Proliferation essential genes (PLEG) of colon cancer cell identified by CRISPR knockout screen database

**Figure 1:**
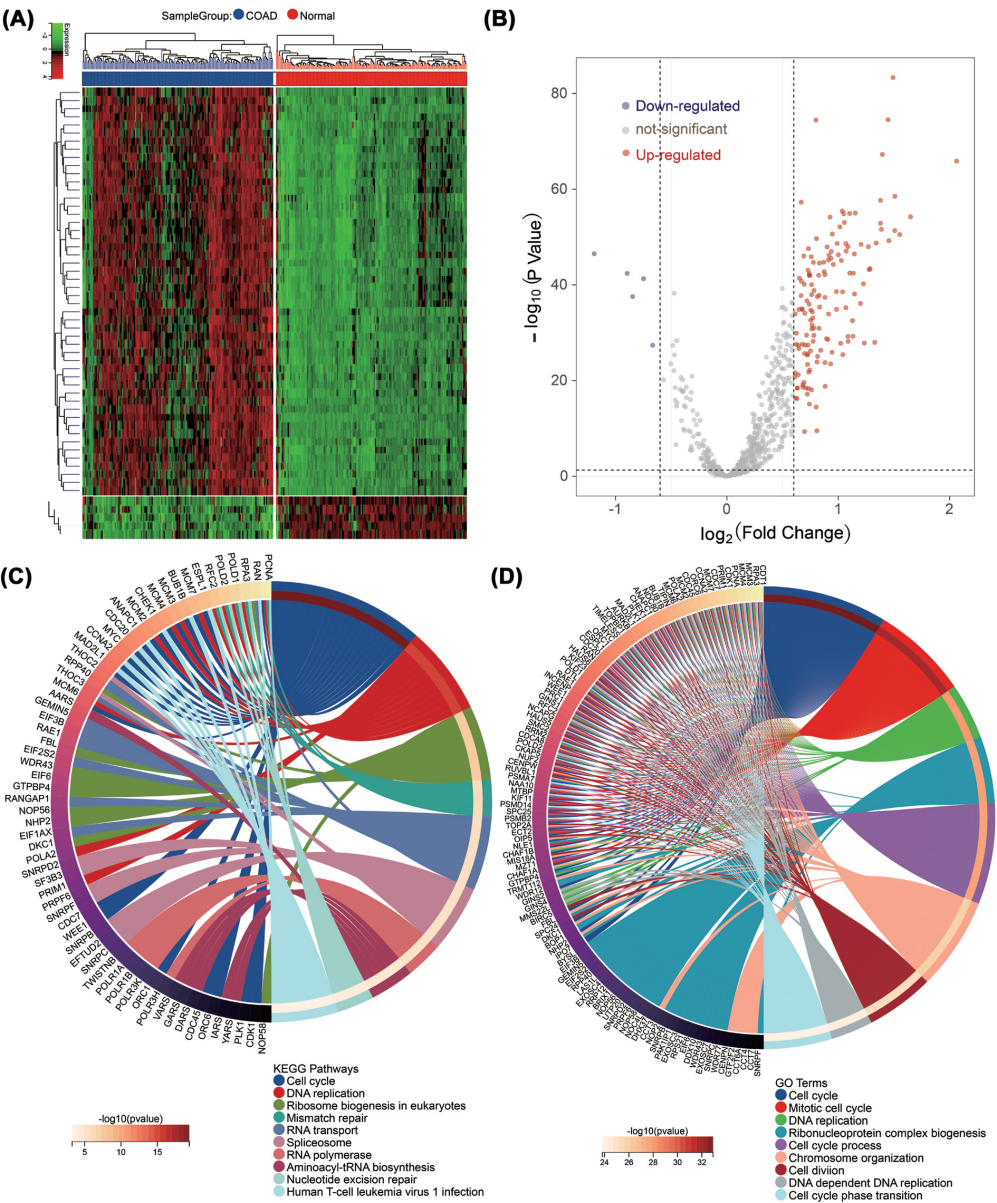
The expression of 730 PLEG in colon cancer (**A**) Heatmap and (**B**) volcano plot was performed to show the differential expression of 730 proliferation essential genes in colon cancer tissues on GSE87211 database. (**C**) KEGG pathways and (**D**) GO-BP terms for the 149 up-regulated proliferation essential genes.

To identify which genes are important for colon cancer cell growth, we looked at CERES scores for colon cancer cell lines in the DepMap database. Genes with a CERES score lower than −1 in over 75% of colon cancer cell lines were considered essential for colon cancer cell growth and were labeled as proliferation essential genes (PLEG) for colon cancer. Upon analysis, we found that there are 730 PLEG in colorectal cancer cells. To further investigate these genes, we analyzed their expression levels in colon cancer tissues and adjacent normal tissues. Because of the significant discrepancy in sample size between colon cancer and normal tissues, the TCGA cohort included 456 colon cancer tissues and only 41 normal tissues. In light of this, the GSE87211 cohort was chosen for investigation. The GSE87211 cohort, which comprises 203 colon cancer tissues and 160 normal tissues, was found to have an ample number of samples with a relatively balanced ratio between colon cancer tissues and normal tissues. The analysis of differentially expressed genes (DEGs) revealed that out of the 730 analyzed PLEG, 149 were significantly up-regulated while only 5 genes were down-regulated (Figure 1A,B). The up-regulated genes were primarily associated with the cell cycle pathway, DNA replication, ribosome biogenesis in eukaryotes, and mismatch repair, as determined by KEGG pathway enrichment analysis (Figure 1C). Furthermore, the GO-BP analysis also revealed that the up-regulated genes were mainly enriched in processes such as cell cycle, mitotic cell cycle, DNA replication, and ribonucleoprotein complex biogenesis (Figure 1D). However, the small number of downregulated genes made it difficult to reliably perform KEGG and GO analysis. These findings imply that the genes that play a crucial role in colon cancer cell proliferation are specifically linked to the cell cycle pathway.

### Construction of co-expressed gene modules and identification of survival-related module

We conducted a weighted gene co-expression network analysis (WGCNA) on the 149 up-regulated PLEG identified in the study. Three WGCNA modules were constructed, namely the blue module, turquoise module, and grey module (which was used for unassigned genes and did not represent a real module), with soft-threshold β = 6 being selected (Supplementary Figure S1A–C). The blue module comprised 68 genes, the turquoise module comprised 78 genes, and the grey module comprised 3 genes. To identify survival-related modules, we correlated module eigengene values with clinical traits of samples using average linkage hierarchical clustering algorithm and Spearman correlation coefficient. Supplementary Figure S1D showed that the blue module exhibited the strongest negative association with survival time; hence, it was identified as the survival-related module. KEGG pathway enrichment analysis revealed that the 68 genes in the blue module were enriched in cell cycle, DNA replication, RNA polymerase, and mismatch repair (Supplementary Figure S1E).

### Construction of proliferation essential genes signature (PLEGs) prognostic model for colon cancer patients

**Figure 2:**
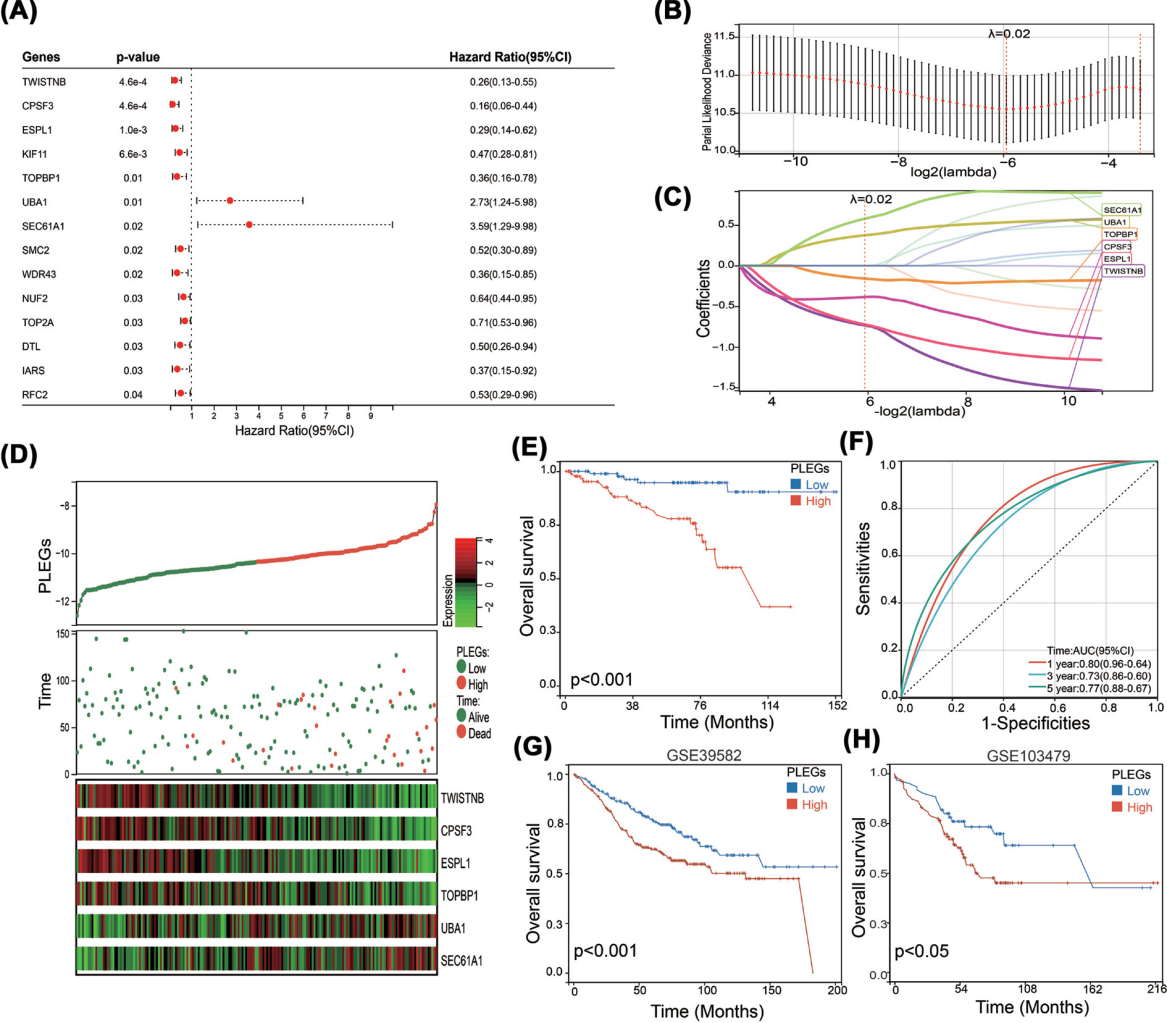
Construction of a PLEGs model for colon cancer patients (**A**) Univariate Cox survival analysis on theses 68 genes in the blue module. (**B,C**) The LASSO-Cox regression model calculated the coefficient and achieved the PLEGs. (**D**) The distribution of survival status, PLEGs, and the heatmap of 6 gene expression levels of each colon cancer patients. (**E**) Kaplan–Meier analysis of the PLEGs model. (**F**) Time-dependent ROC curves for survival prediction. (**G**) Kaplan–Meier analysis of the PLEGs model in GSE39582 cohort. (**H**) Kaplan–Meier analysis of the PLEGs model in GSE103479 cohort.

In order to assess the predictive significance of the 68 genes in the blue module, we carried out univariate Cox proportional hazards regression analysis on the GSE87211 cohort. The outcomes of this analysis indicated that elevated expression levels of *ESPL1* (Extra Spindle Pole Bodies Like 1, Separase), *KIF11* (Kinesin Family Member 11), *TWISTNB*, *CPSF3* (Cleavage and Polyadenylation Specific Factor 3), *TOPBP1* (DNA Topoisomerase II Binding Protein 1), *SMC2* (Structural Maintenance Of Chromosomes 2), *WDR43* (WD Repeat Domain 43), *NUF2* (NUF2 Component Of NDC80 Kinetochore Complex), *TOP2A* (DNA Topoisomerase II Alpha), *DTL* (Denticleless E3 Ubiquitin Protein Ligase Homolog), *IARS* (Isoleucyl-TRNA Synthetase 1), and *RFC2* (Replication Factor C Subunit 2) were correlated with a more favorable prognosis in patients suffering from colon cancer. Additionally, high expression of *UBA1* and *SEC61A1* were associated with a poorer prognosis in colon cancer patients (Figure 2A). By conducting univariate Cox regression survival analysis, we selected 14 genes for further LASSO regression analysis. The partial likelihood deviance versus log(λ) was plotted in Figure 2B, and a value of 0.02 for λ was selected using 10-fold cross-validation. The PLEGs prognostic model was then derived. PLEGs = (−0.7350 × expression level of *TWISTNB*) + (−0.725 × expression level of *ESPL1*) + (−0.159 × expression level of *TOPBP1*) + (−0.386 × expression level of *CPSF3*) + (0.375 × expression level of *UBA1*) + (0.579 × expression level of *SEC61A1*) (Figure 2C). The study presented in Figure 2D investigated the relationship between the mRNA levels of several genes, including *TWISTNB, ESPL1, TOPBP1, C2PSF3, UBA1*, and *SEC61A1*, and the survival of colon cancer patients. The results showed that patients with a PLEGs-low subtype had longer survival time than those with a PLEGs-high subtype, as demonstrated by the Kaplan–Meier survival analysis in Figure 2E. Moreover, time-dependent ROC analysis was used to evaluate the predictive potential of the PLEGs prognostic prediction model. The AUC of the model for overall survival was 0.80 at 1 year, 0.73 at 3 years, and 0.77 at 5 years (Figure 2F), indicating that the model had a good predictive ability. These findings were further supported by the external validation cohorts of GSE39582 and GSE103479, which also showed that the PLEGs-low subtype had better overall survival than the PLEGs-high subtype (Figure 2G,H). Overall, the results of the study suggest that PLEGs may serve as a valuable prognostic marker for colon cancer patients.

To further evaluate the reliability of PLEGs prognostic prediction model, we categorized colon cancer patients based on several factors such as sex, age, stage, tumor location, *Braf* mutation, *Kras* mutation, and *Tp53* mutation. After categorizing colon cancer patients by specific factors such as male sex (Supplementary Figure S2A), age ≤60 (Supplementary Figure S2C), age >60 (Supplementary Figure S2D), stage 1&2 (Supplementary Figure S2E), stage 3&4 (Supplementary Figure S2F), distal tumor location (Supplementary Figure S2G), proximal tumor location (Supplementary Figure S2H), no *Braf* mutation (Supplementary Figure S2I), no *Kras* mutation (Supplementary Figure S2K), *Tp53* mutation absence (Supplementary Figure S2M), and presence (Supplementary Figure S2N), PLEGs continued to be a significant independent prognostic marker, with colon cancer patients diagnosed as PLEGs-high subtype exhibiting inferior prognosis. The PLEGs model was found to be an insignificant predictor for colon cancer patients with female sex (Supplementary Figure S2B), *Braf* mutation positive (Supplementary Figure S2J), and *Kras* mutation positive (Supplementary Figure S2L). It is well-documented that gender is an independent prognostic factor for colon cancer, with females exhibiting better prognosis than males [20]. However, the underlying biological mechanisms responsible for gender-specific differences in colon cancer survival remain unknown. This suggests that the ineffectiveness of the PLEGs model in predicting survival in female patients may be attributed to their significantly better prognosis. In summary, these results further establish the reasonable ability of the PLEGs prognostic prediction model for stratification.

### PLEGs predicted the response to chemotherapy in colon cancer patients

**Figure 3:**
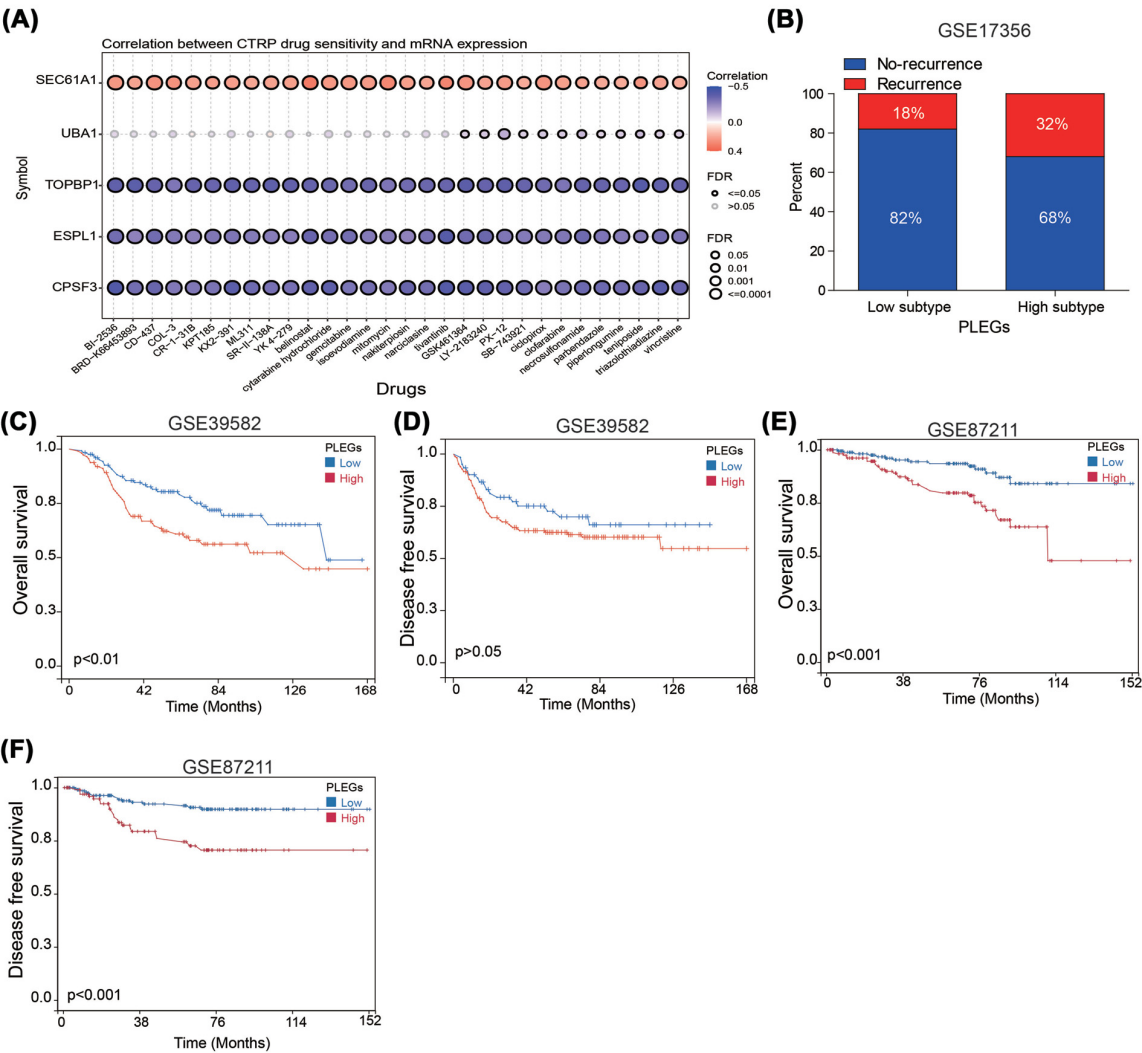
The correlation between PLEGs and chemotherapy response in colon cancer patients (**A**) The correlation between GDSC drug sensitivity and PLEG expression. (**B**) The correlation of PLEGs and recurrence after chemotherapy. (**C**) Survival analysis reveals difference in overall survival of colon cancer patients treated with chemotherapy in GSE39582. (**D**) Survival analysis reveals difference in disease free survival of colon cancer patients treated with chemotherapy in GSE39582. (**E**) Survival analysis reveals difference in overall survival of colon cancer patients treated with chemotherapy in GSE87211. (**F**) Survival analysis reveals difference in disease free survival of colon cancer patients treated with chemotherapy in GSE87211.

Research conducted previously has consistently demonstrated the significant benefits of administering adjuvant chemotherapy in conjunction with surgical treatment for patients with colon cancer. Despite these benefits, drug resistance presents a major challenge in achieving positive treatment outcomes. To explore potential mechanisms of drug resistance, we conducted an analysis of the Cancer Therapeutics Response Portal (CTRP) database to investigate the relationship between drug sensitivity and the expression of PLEGs genes. Our findings indicate that higher levels of SEC31A1 are positively associated with increased IC50 values of cancer therapy drugs, whereas the expression of TOPBP1, ESPL1, and CPSF3 displayed an opposite correlation (Figure 3A). These results suggest that these findings could have important implications in guiding the development of chemotherapy regimens for clinical use. It is worth noting that within the PLEGs-low subtype, 18% of colon cancer patients experienced recurrence post-chemotherapy treatment. Comparatively, within the PLEGs-high subtype, the recurrence rate was 32% post-chemotherapy treatment (Figure 3B). Additionally, we investigated the correlation between PLEGs and the response to adjuvant chemotherapy. Survival analysis demonstrated that patients belonging to the PLEGs-low subtype experienced greater benefits in terms of overall survival (OS) and disease-free survival (DFS) in GSE39582 cohort (Figure 3C,D). Unfortunately, there were no significant differences observed in DFS. Additionally, we found that patients classified as PLEGs-Low subtype in the GSE87211 cohort also exhibited significantly better OS and DFS compared to patients classified as PLEGs-High subtype (Figure 3E,F). In summary, PLEGs proved to be a reliable tool for predicting the response of colon cancer patients to chemotherapy, with individuals classified under the PLEGs-low subtype potentially reaping significant benefits from adjuvant chemotherapy.

### Construction of a clinical prognostic prediction model

**Figure 4:**
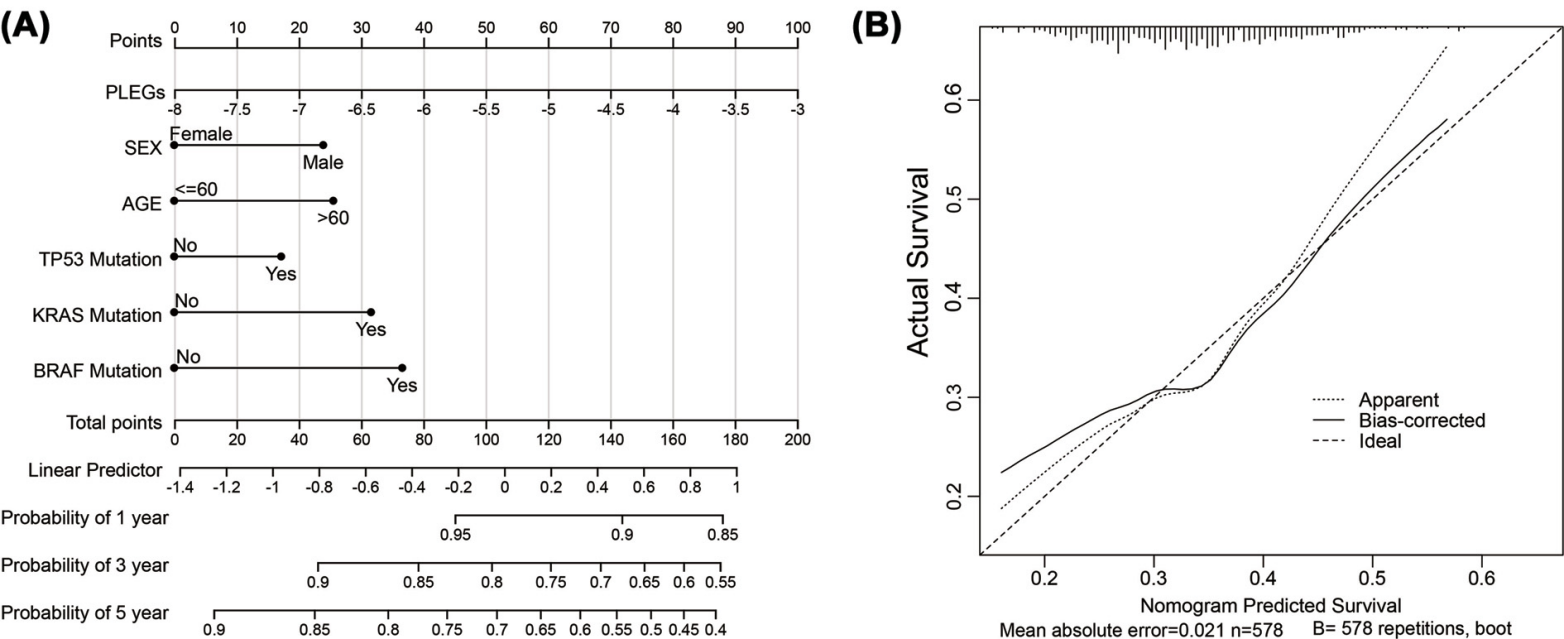
Construction of the clinical prognostic prediction model (**A**) The survival nomogram was built to calculate the 1-, 3-, and 5-year survival rate based on 6 variables containing PLEGs, sex, age, *Tp53* mutation, *Kras* mutation and *Braf* mutation. (**B**) The comparison between the ideal model and standard curve.

A statistical predictive model has been simplified and made more intuitive through the creation of a clinical nomogram, which serves as a reliable tool for generating a simple graph. The nomogram is designed to facilitate score calculation for six key clinical indicators: PLEGs, sex, age, *Tp53* mutation, *Kras* mutation, and *Braf* mutation. Each factor is assigned a weighted point value that reflects its impact on a patient’s risk of survival. Patients with higher total scores are considered to have a poorer prognosis and a higher risk of death. The nomogram predicts the probability of survival at 1-, 3-, and 5-year intervals for colon cancer patients (Figure 4A), and its accuracy is demonstrated through good agreement in the calibration plots (mean absolute error = 0.021, Figure 4B).

### *UBA1* was high expression in colon cancer

**Figure 5:**
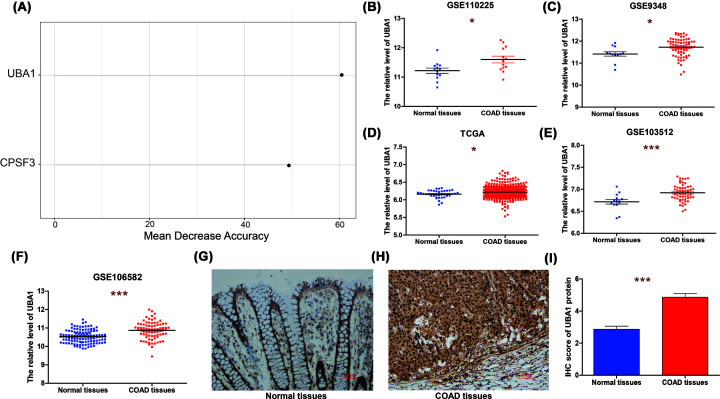
UBA1 was high expression in colon cancer (**A**) Random Forest feature importance ranking for the PLEG. (**B–F**) The expression of *UBA1* between colon cancer tissues and normal tissues based on (B) GSE110225, (C) GSE9348, (D) TCGA, (E) GSE103512 and (F) GSE106582. (**G,H**) Representative IHC images of UBA1 protein expression in (G) normal tissues and (H) colon cancer tissues. (**I**) IHC score for UBA1 in colon cancer patients; *, *P*<0.05; ***, *P*<0.001.

Subsequently, the importance of the 6 PLEGs genes in distinguishing between normal and colon cancer tissues was assessed via random forest analysis. Remarkably, UBA1 emerged as the most crucial gene among them, as illustrated in Figure 5A. In order to further scrutinize the expression of UBA1 in colon cancer, the UBA1 mRNA expression data was sourced from several databases such as TCGA and GEO (GSE110225, GSE9348, GSE103512, and GSE106582). The study included a total of 13 colon cancer tissues and 13 corresponding normal tissues from GSE110225, while GSE9348 contained 70 colon cancer tissues and 12 normal tissues, GSE103512 consisted of 55 colon cancer tissues and 14 normal tissues, and GSE106582 encompassed 77 colon cancer tissues and 117 normal tissues. Intriguingly, results from all the five cohorts unequivocally revealed a significant overexpression of UBA1 mRNA levels in colon cancer tissues, as depicted in Figure 5B–F.

Meanwhile, we also conducted an IHC analysis to evaluate the protein expression levels of UBA1. Our findings revealed a significant increase in UBA1 protein expression in 30 colon cancer tissues when compared to their paired adjacent normal tissues (Figure 5G–I). This suggests that both UBA1 mRNA and protein expression levels are higher in colon cancer tissues when compared to normal colon tissues.

### Knockdown of *UBA1* suppressed proliferation, migration and invasion of colon cancer cells

**Figure 6:**
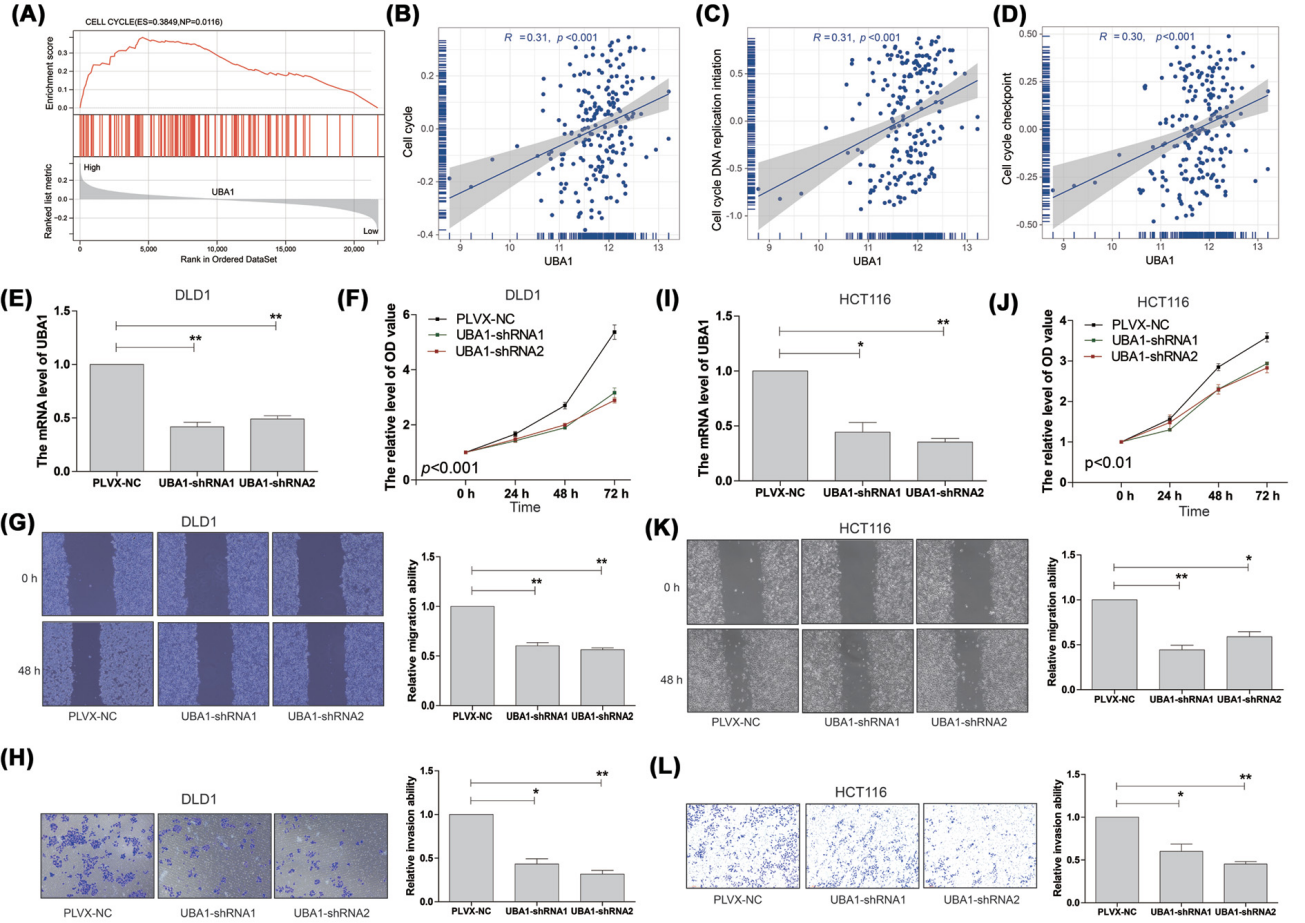
Knockdown *UBA1* suppressed proliferation, migration and invasion of colon cancer cells (**A**) GSEA analysis indicated that the high expression of *UBA1* is related to the cell cycle pathway. (**B–D**) Correlation analysis of *UBA1* and (B) cell cycle, (C) cell cycle DNA replication initiation and (D) cell cycle checkpoint. (**E**) The mRNA expression of *UBA1* in *UBA1* knockdown DLD1 cells. (**F–H**) Knockdown *UBA1* suppressed (F) proliferation, (G) migration, and (H) invasion of DLD1 cells. (**I**) The mRNA expression of *UBA1* in *UBA1* knockdown HCT116 cells. (**J–L**) Knockdown *UBA1* suppressed (J) proliferation, (K) migration, and (L) invasion of HCT116 cells; *, *P*<0.05; **, *P*<0.01.

Next, the GSEA method was employed to identify differentially activated pathways in colon cancer with high *UBA1* expression. The results revealed a positive correlation between high *UBA1* expression and the cell cycle pathway (Figure 6A). Additionally, GSVA was conducted to assess the relationship between *UBA1* and cell cycle-related pathways. The findings indicated a positive correlation between UBA1 and the cell cycle (Figure 6B), cell cycle DNA replication initiation (Figure 6C), and cell cycle checkpoint (Figure 6D). To decipher the biological functions of *UBA1* in colon cancer, the effects of decreased *UBA1* expression on DLD1 and HCT116 cell proliferation, migration, and invasion were investigated. The CCK8 assay demonstrated a significant attenuation of cell proliferation following *UBA1* knockdown in DLD1 and HCT116 cells (Figure 6E,F,I,J). Furthermore, cell migration assay revealed that *UBA1* knockdown significantly suppressed the migration of DLD1 and HCT116 cells (Figure 6G,K). Transwell assay showed that *UBA1* knockdown suppressed the invasion abilities of DLD1 and HCT116 cells (Figure 6H,L).

## Discussion

Although there has been some advancement in the prompt detection and management of colon cancer, local recurrence and metastasis after surgery continue to represent the primary reasons for mortalities in patients with this condition. Thus, identifying prospective targets for the diagnosis and therapy of colon cancer is critical in a clinical setting to ameliorate the prognoses of individuals suffering from this disease [21–24]. To identify potential cancer therapeutic targets, the DepMap database was established. Initially, the Broad institute used RNAi technology to measure gene dependencies. Later, with the rise and wide application of CRISPR-Cas9 technology, the Broad institute and the Sanger institute used CRISPR-Cas9 technology to determine the gene dependencies [9]. The discovery of genes that are crucial for proliferation in colon cancer will provide fresh perspectives on tumorigenesis and potential avenues for therapeutic intervention.

The study aimed at identifying potential targets for the diagnosis and treatment of colon cancer. To achieve this, we identified a set of 730 genes that are essential for the proliferation of colon cancer cells. We then compared the expression levels of these genes in colon cancer tissues and normal tissues using the GSE87211 database, and found that 149 genes were up-regulated in colon cancer tissues. Further gene enrichment analysis revealed that these up-regulated genes were primarily involved in the cell cycle-related signaling pathway in colon cancer tissues.

Based on WGCNA, univariate cox regression survival analysis and Lasso analysis, we established a PLEGs model (including *TWISTNB*, *ESPL1*, *TOPBP1*, *CPSF3*, *UBA1*, and *SEC61A1*) screened from these proliferation essential genes, and this model could divide colon cancer patients into the low-risk group and high-risk group. Meanwhile, the PLEGs model was validated in the GSE39582 and GSE103479 database. In addition, our research has yielded significant findings regarding the use of PLEGs as a predictive tool for chemotherapy treatment in colon cancer. Specifically, we have found that patients classified under the PLEGs-Low subtype exhibit a more favorable prognosis after receiving chemotherapy, in comparison to those classified under the PLEGs-High subtype. Notably, the recurrence rate among colon cancer patients in the PLEGs-Low subtype who underwent chemotherapy was significantly lower than in the PEGs-high subtype. Given these findings, we believe that PLEGs could prove to be a valuable instrument in predicting outcomes of chemotherapy treatment in colon cancer.

By utilizing random forest analysis on six PLEG, *UBA1* was determined to be the gene with the highest significance in distinguishing between colon cancer tissues and healthy tissues. This suggests that *UBA1* may play a pivotal role in the progression of colon cancer. *UBA1* is the major E1 enzyme that initiates ubiquitylation, responsible for initiating the transfer of ubiquitin molecules to target proteins, which are to be degraded by proteasomes [25]. Protein ubiquitination has been shown to regulate a variety of cellular processes, including cell cycle progression, apoptosis, and protein trafficking. Previous studies in yeast and mammalian cells have shown that disrupting E1 enzyme function leads to cell cycle arrest. The results of survival analysis showed that the expression of *UBA1* may be related to the prognosis of cancer. The high expression of *UBA1* is associated with poor prognosis of liver cancer patients. *UBA1* knockdown inhibited proliferation, migration, and invasion of liver cancer cells. However, high expression of *UBA1* is associated with good prognosis in prostate cancer patients [26]. However, the biological role of *UBA1* is less well understood in colon cancer. In the study, the expression level of *UBA1* was found to be strikingly upregulated in colon cancer. The silencing of *UBA1* has been shown to inhibit the proliferation, migration, and invasion of colon cancer cells. Thus, *UBA1* is an independent indicator for colon cancer progression.

However, it is important to acknowledge several limitations of this study. First, functional validation of the identified PLEGs and UBA1 in colon cancer was limited, and further experimental studies are needed to confirm their roles in tumorigenesis and progression. Second, while external validation cohorts were used, additional independent validation studies are needed to further validate the prognostic value and clinical applicability of the PLEGs model. Despite these limitations, the findings of the present study provide valuable insights into potential targets for the diagnosis, prognosis, and treatment of colon cancer.

## Conclusion

Our findings suggest that PLEGs can be employed as a tool for patient stratification and identification of those with a higher likelihood of responding well to chemotherapy treatment. Additionally, we have uncovered evidence supporting the pivotal role of *UBA1* in the onset and progression of colon cancer. The present study offers novel perspective into the underlying mechanisms of colon cancer, and has the potential to inform the development of preventive and therapeutic interventions targeting this disease.

## Supplementary Material

Supplementary Figures S1-S2

## Data Availability

The data that support the findings of the present study are openly available in GEO (https://www.ncbi.nlm.nih.gov/geo/) and TCGA (https://portal.gdc.cancer.gov/).

## Abbreviations

CTRP: Cancer Therapeutics Response Portal
DEG: different expression gene
DFS: disease-free survival
OS: overall survival
PLEG: proliferation essential gene
WGCNA: weighted gene co-expression network analysis
