# Supplementary material for: Prognostic value and chemotherapy response prediction of a proliferation essential gene signature in colon cancer
Source: Biosci Rep. 2023 Jul 19;43(7):BSR20230733. doi: 10.1042/BSR20230733 (PMC10357000; doi:10.1042/BSR20230733)
Supplement: Supplementary Figures S1-S2 [file BSR-2023-0733_supp.pdf]

Supplementary Figures

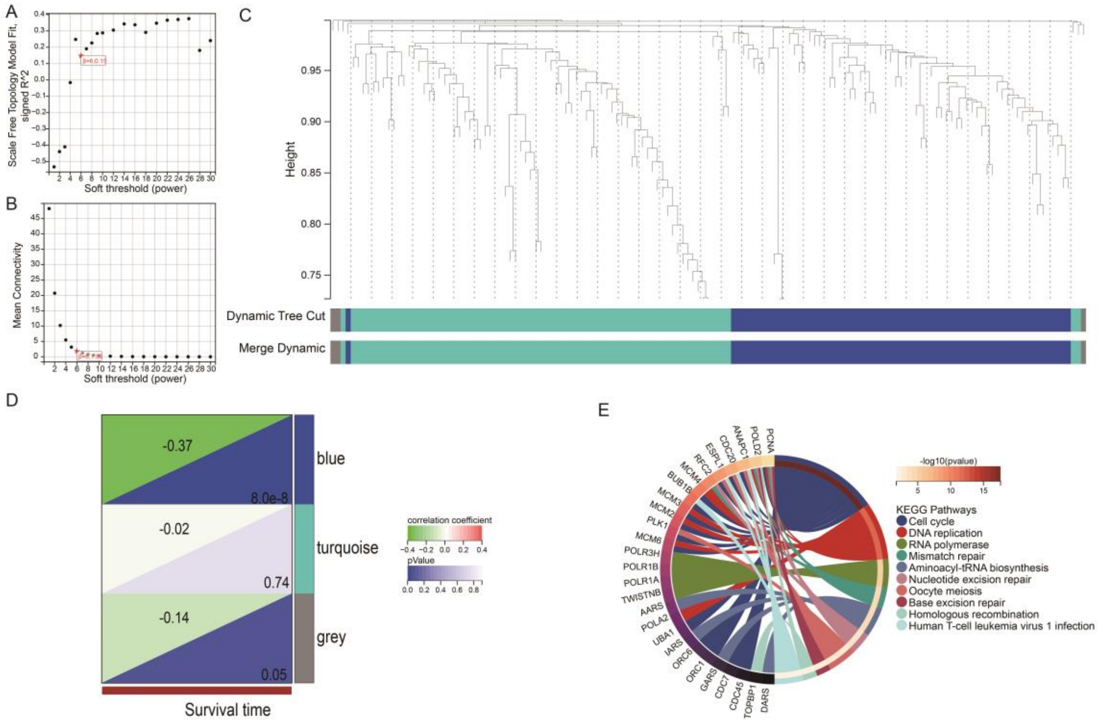

Supplementary Figure 1. Identifying co-expression module genes using the WGCNA.

(A) Clustering dendrogram of colon cancer tissues based on the GSE87211 database. (B) Relationship between scale-free topology model fit and soft thresholds. (C) Dendrogram of modules identified by WGCNA. (D) Correlation of WGCNA modules with clinical outcomes. (E) KEGG pathway analysis for genes within blue modules.

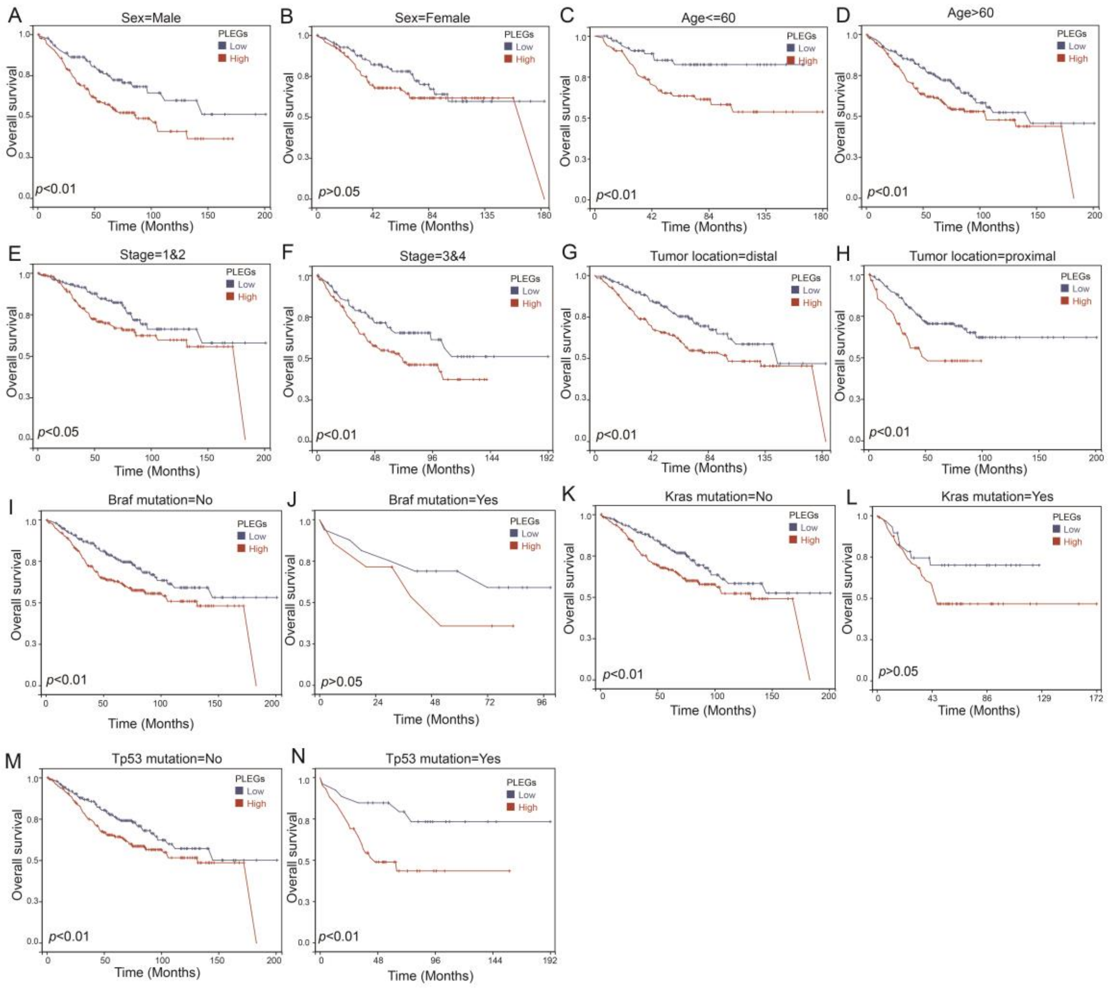

Supplementary Figure 2. Kaplan–Maier survival analysis of colon cancer patients according to PLEGs model in different subgroups.

(A&B) Survival analysis of colon cancer patients with (A) sex=male and (B) sex=female. (C&D) Survival analysis of colon cancer patients with (C) age<=60 and (D) age>60. (E&F) Survival analysis of colon cancer patients with (E) stage=1&2 and (F) stage=3&4. (G&H) Survival analysis of colon cancer patients with (G) tumor location=distal and (H) tumor location= proximal. (I&J) Survival analysis of colon cancer patients with (I) *Braf* mutation= no and (J) *Braf* mutation= yes. (K&L) Survival analysis of colon cancer patients with (K) *Kras* mutation= no and (L) Kras mutation= yes. (M&N) Survival analysis of colon cancer patients with (M) *Tp53* mutation= no and (N) *Tp53* mutation= yes.
